# Simultaneous multi-analyte urinary protein assay for bladder cancer detection

**DOI:** 10.1186/1472-6750-14-24

**Published:** 2014-04-01

**Authors:** Charles J Rosser, Yunfeng Dai, Makito Miyake, Ge Zhang, Steve Goodison

**Affiliations:** 1 MD Anderson Cancer Center Orlando, Orlando, FL, USA; 2 Nonagen BioScience Corp, Jacksonville, FL, USA; 3 University of Hawaii Cancer Center, Honolulu, HI, USA; 4 Department of Biostatistics, The University of Florida, Gainesville, FL, USA; 5 Department of Health Sciences Research, Mayo Clinic, Jacksonville, FL, USA

**Keywords:** Biomarkers, Bladder cancer, Q-plex, Protein, Urine

## Abstract

**Background:**

The ability to accurately measure multiple proteins simultaneously in a single assay has the potential to markedly improve the efficiency of a myriad of clinical assays. Here, we tested the performance of a new, multiplex protein array platform to quantitate three bladder cancer-associated proteins in urine samples. The following analytes, interleukin 8 (IL8), matrix metallopeptidase 9 (MMP9), and vascular endothelial growth factor A (VEGFA) were monitored using Q-plex, a customized multiplex ELISA system from Quansys Biosciences, and individual target commercial ELISA kits. The performance of the two approaches was compared by evaluating the diagnostic accuracy of the biomarker assays in samples from a cohort of 73 subjects of known bladder cancer status.

**Results:**

The combination biomarker panel analyses revealed an AUROC value of 0.9476 for the Q-plex assay, and 0.9119 for the combination of the single-target ELISA assays. The Q-plex assay achieved an overall diagnostic sensitivity of 0.93 and specificity of 0.81, and the individual target ELISA assays achieved an overall sensitivity of 0.77 and specificity of 0.91.

**Conclusion:**

Based on these encouraging preliminary data, we believe that the Q-Plex technology is a viable platform that can be exploited as an efficient, highly accurate tool to quantitate multiplex panels of diagnostic proteins in biologic specimens.

## Practical applications

While there continues to be an increasing number of biomarker discovery studies published, very few new cancer biomarkers have entered the clinic over the past 30 years. One reason for this has been the reliance on assays of single biomarkers for the evaluation of cancers that have a broad spectrum of molecular changes. Coupled with the observed variation between individuals and the heterogeneity within tumors, there has been a necessary shift to molecular signatures comprised of multiple biomarkers. Technologies that can efficiently, simultaneously monitor molecular signatures would be pivotal in moving promising biomarker panels towards clinical utility. Quansys Biosciences have developed the Q-Plex, a multiplex ELISA system that prints capture antibodies as an array in a single well. In this study, we showed that this system could simultaneously and accurately monitor three target proteins in urine samples. Multiplexed ELISAs such as the Q-plex system can maximize data generation from each sample, and provide a low cost per data point. The further optimization and development of robust Q-plex assays in complex biological fluids will allow this technology to become more widely adopted.

## Background

The advent of advanced molecular profiling techniques has enabled the derivation of molecular signatures that hold promise for more accurate, even individualized patient evaluation [1]. A number of molecular signature assays are now being incorporated into clinical practice [2,3], but the assays employed to monitor multiple targets per sample are, to date, rather complex and thus, expensive, and often require centralized processing and analysis. We have previously employed a range of proteomic [4,5] and genomic [6,7] technologies to profile naturally voided urine samples with the aim of identifying disease-associated biomarkers that could be developed for the non-invasive detection of bladder cancer.

The objective of the current feasibility study was to test the ability of a customized multiplex assay system to accurately and simultaneously monitor three urinary protein biomarkers from one of our validated bladder cancer signatures [8]. The levels of the three proteins, interleukin 8 (IL8), matrix metallopeptidase 9 (MMP9), and vascular endothelial growth factor A (VEGFA), were quantitated in real-world urine samples and the diagnostic performance of the multiplex system was compared with analyses performed using three individual target commercial ELISA kits.

The multiplex assay used Q-Plex™ technology (Quansys BioScience, Logan, UT), which involves the micro-spotting of individual groups of capture antibody in either a cartesian or polar coordinate system on the bottom of a 96 well plate with each spot being its own ‘micro ELISA’ assay [9]. Micro-spotted systems have the advantage of higher assay sensitivities and faster reaction kinetics due to minimizing diffusion constraints for analyte/antibody binding [10]. Standard ELISA incubation steps such as initial sample incubation, washing, secondary antibody incubation, washing, incubation are employed, but the labeling and reporting system used in the Q-Plex Array™ is chemiluminescent. Chemiluminescent ELISAs have been shown to be more sensitive than colorimetric detection systems [11,12].

In our study, the multiplex ELISA assay was shown to be highly sensitive and to be consistently accurate across wells and plates. Most importantly, the assay achieved comparable diagnostic performance with respect to bladder cancer detection as the individual target commercial ELISA assays [13-17]. The system can be applied to a range of biological biomarker monitoring applications, and we are working to expand the multiplex platform to provide a high-throughput approach to quantitatively detect more comprehensive bladder cancer-associated diagnostic protein signatures in voided urine samples.

## Results and discussion

### Q-plex assay characterization

The physical components, a library of capture antibodies, and the secondary reagents of the Q-Plex™ system have undergone extensive optimization for consistent implementation [18,19]. Ranges for each analyte assay were evaluated by dilution of standards to determine upper ranges where high-end hook effect and apparent antibody saturation are avoided and lower ranges that are above detection limits. Lower limits of detection (LLD) were calculated based on 2× the standard deviation of the background of 20 negative wells. LLD for IL8, MMP9 and VEGFA were 0.4 pg/ml, 297 pg/ml and 0.5 pg/ml, respectively, demonstrating sufficient sensitivity to detect proteins present in small amounts in voided urine samples. The LLD data for the individual ELISA kits (1.02 pg/ml for IL8, 66.1 pg/ml for MMP9, and 23.7 pg/ml for VEGFA) were comparable in range for the most part. Any differences are presumably due to the antigen binding characteristics of the proprietary antibodies. Intra assay precision was measured with acceptance criteria of a coefficient of variation (%CV) of less than 15.0. Median inter assay variability across all plates was also determined to be less than 15% CV for each analyte. As the technology is an array, all components were checked for cross reactivity with other components in the antigen and antibody cocktails and confirmed to have less than 0.5% cross reactivity. Lower limits of quantification (LLOQ) were determined to be the lowest point of the 10-point positive standard curve where the back-fit regression values were within 20% of the known value. The minimal LLOQ, maximum and average LLOQ for IL8, MMP9, and VEGFA were 0.3 pg/ml, 0.7 pg/ml, and 0.7 pg/ml; 35373 pg/ml, 52433 pg/ml, and 40782 pg/ml; and 19 pg/ml, 613 pg/ml, and 419 pg/ml, respectively. Only three of the 73 urine samples subsequently tested (two for IL8 and one for VEGFA) fell outside the standard curve and required extrapolation.

### Urine sample analysis

**Figure 1:**
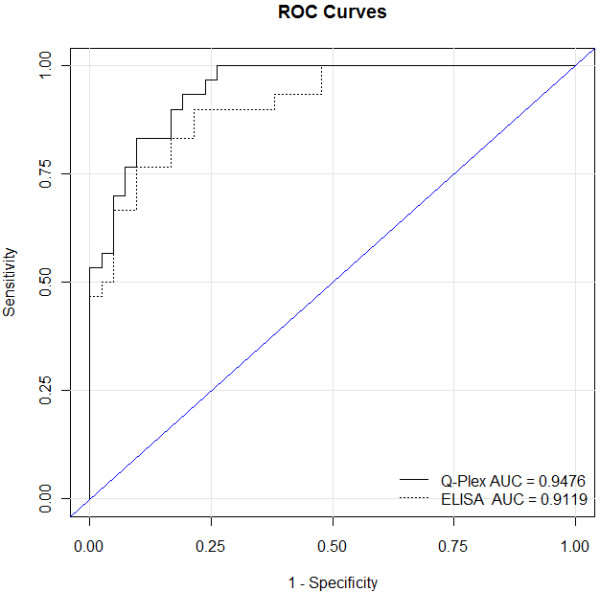
**Diagnostic performance of bladder cancer-associated molecular panel comprised of three biomarkers.** ROC curves were plotted to illustrate the performance characteristics of the 3-biomarker signature for the detection of bladder cancer in urine samples using the Q-Plex multiplex assay (solid line) and commercial ELISA assays (dotted line). Based on the area under the ROC curve (AUROC), Youden Index cutoff values that maximized the sum of sensitivity and specificity were determined for the combination of biomarkers. The Q-Plex multiplex assay achieved an overall sensitivity of 0.93 and specificity of 0.81 (AUROC 0.9476). The combination of data from the individual target ELISA assays achieved an overall sensitivity of 0.77 and specificity of 0.91 (AUROC 0.9119). Thus, the comparison of multiplex results with standard ELISA of these three diagnostic biomarkers showed similar results and trends.

After confirming the robustness of the multiplex assay for these specific biomarkers, it was used to test a voided urine sample set. Seventy-three clinical urine samples from MD Anderson Cancer Center Orlando were made available for analysis. Demographics and disease characteristics of the entire cohort are summarized in Table 1. The ability of each of the test biomarkers within the multiplex array to predict the presence of bladder cancer was analyzed using nonparametric ROC analyses, according to National Cancer Institute guidelines [20]. Urinary IL8 was the most accurate single biomarker for bladder cancer detection with an AUROC of 0.907 (95% CI: 0.830 - 0.985), a sensitivity of 90%, specificity of 86%, PPV of 82% and NPV of 92%. Urinary MMP9 data generated an AUROC of 0.533 (95% CI: 0.392 - 0.674), sensitivity of 45%, specificity of 76%, PPV of 58% and NPV of 65%, and VEGFA as an individual analyte was noted to have an AUROC of 0.524 (95% CI: 0.386 - 0.661), sensitivity of 17%, specificity of 95%, PPV of 71% and NPV of 61%. Through combinatorial analysis of all 3 biomarkers using optimal cutoff values defined by Youden index calculations, the AUROC (Figure 1) for the diagnostic panel using the multiplex array was 0.9476 [95% CI: 0.903 - 0.992]. The combination assay achieved an overall sensitivity of 0.93, specificity of 0.81, PPV of 0.78 and NPV of 0.94 for bladder cancer classification.

**Table 1:** Demographic and clinicopathologic characteristics of 73 subjects comprising study cohort

	**Bladder cancer**	**Benign controls**
	**n = 31**	**n = 42**
**Median Age (range, y)**	**71 (54–93)**	**62 (24–81)**
**Male : Female ratio**	**26:5**	**35:7**
**Suspicious/positive cytology**	**32%**	**0%**
**Clinical stage**		
**Tis**	**3**	**N/A**
**Ta**	**12**	**N/A**
**T1**	**2**	**N/A**
≥**T2**	**14**	**N/A**
**Tumor grade**		
**Low**	**4**	**N/A**
**High**	**27**	**N/A**

Spearman Correlation coefficient was not lower than 0.417 for any biomarker, thus comparison of the results of the Q-Plex™ assay to those of the commercial ELISA kits directed towards IL8, MMP9 and VEGFA were encouraging (Tables 2 &3). The ability of each of the test biomarkers of the individual commercial ELISA assays to predict the presence of bladder cancer was analyzed using nonparametric ROC analyses. As with the multiplex assay, urinary IL8 was the most accurate single biomarker when monitored by individual ELISA with an AUROC of 0.878 (95% CI: 0.783 - 0.972), a sensitivity of 87%, specificity of 86%, PPV of 82% and NPV of 90%. Urinary MMP9 data generated an AUROC of 0.548 (95% CI: 0.409 - 0.688), sensitivity of 45%, specificity of 76%, PPV of 58% and NPV of 65%, while VEGFA was noted to have an AUROC of 0.493 (95% CI: 0.356 - 0.630), sensitivity of 16%, specificity of 95%, PPV of 71% and NPV of 61%. Through combination of data from all 3 biomarkers, the AUROC for the diagnostic panel from the ELISA assays was 0.9119 [95% CI: 0.796 - 0.968], with an overall sensitivity of 0.77, specificity of 0.91, PPV of 0.86 and NPV of 0.84 for bladder cancer classification. Thus, the comparison of multiplex results with standard ELISA of these three diagnostic biomarkers showed similar results and trends.

**Table 2:** Comparison of Q-plex™ technology to traditional ELISA assays

	**Q-plex™ array**		**Commercial ELISA assay**	
	**Cancer**	**Benign**	**Cancer**	**Benign**
**IL8 (pg/ml)**				
**Median**	127.7	2.2	100.8	144.3
**(Min, Max)**	(0.4, 5,087.5)	(0.5, 185.3)	(0, 4,355.3)	(0, 506.3)
Mean ± SD*	423.6 ± 931.2	14.6 ± 40.9	276.8 ± 768.9	180.0 ± 138.2
**MMP9 (pg/ml)**				
**Median**	268,688	248,730.5	2,833.2	545.3
**(Min, Max)**	(176,103.9, 3,783,990.1)	(162,655.6, 867,918.5)	(0, 12,988.9)	(0, 7,146)
**Mean + SD**	469,075.4 ± 655,960.4	320,746.6 ± 171,092.6	5,236.0 ± 4,144.5	876.5 ± 1,353.0
**VEGFA (pg/ml)**				
**Median**	12,599.4	12,677.5	143.6	19.5
**(Min, Max)**	(2,093.3, 498,114.9)	(810.1, 262,356.7)	(1.1, 9,874.1)	(0, 184.2)
**Mean + SD**	33,483.5 ± 88,403.7	20,690.5 ± 39,702.7	1061.6 ± 2132.9	36.2 ± 47.4

*, *p* < 0.05 comparing cancer cohort to benign cohort for both Q-Plex™ Array and Commercial ELISA Assay.

**Table 3:** **Performance comparison of Q-plex**™ **and individual-target ELISA assays for the detection of bladder cancer in urine samples**

**Urinary analytes**	**Assay platform**	**AUROC [95% CI]**	**Sensitivity (%)**	**Specificity (%)**	**PPV (%)**	**NPV (%)**
**IL8**	Q-Plex™ Array	0.907 [0.830 - 0.985]	90	86	82	92
	Commercial ELISA	0.878 [0.783 - 0.972]	87	86	82	90
**MMP9**	Q-Plex™ Array	0.533 [0.392 - 0.674]	45	76	58	65
	Commercial ELISA	0.548 [0.409 - 0.688]	45	76	58	65
**VEGFA**	Q-Plex™ Array	0.524 [0.386 - 0.661]	17	95	71	61
	Commercial ELISA	0.493 [0.356 - 0.630]	16	95	71	61
**Combination**	Q-Plex™ Array	0.948 [0.903 - 0.992]	93	81	78	94
	Commercial ELISA	0.912 [0.796 - 0.968]	77	91	86	84

The Quansys Multiplex ELISA is now being optimized to measure the remaining proteins of our highly accurate bladder cancer-associated diagnostic panel comprised of 10 biomarkers [8]. We are currently producing specific antibodies (Nonagen BioScience) for the 10 analytes in our diagnostic panel, which can potentially be incorporated into a Quansys BioScience custom array system for an integrated bladder cancer detection assay. The ability to analyze multiple proteins per well, in addition to dozens of samples per microplate, results in marked improvements in efficiency and considerable cost savings compared to single analyte ELISA.

Our study has several limitations. Notably, the study cohort of 73 subjects was relatively small, and only three targets from our validated 10-biomarker panel were tested in this study. However this is a proof-of-principle study in which we investigated the potential for multiplexing analyte detection in real-world urine samples. Lastly, as part of a biomarker discovery and validation program, archived urines were retrieved from tissue banks for analysis. Prolonged storage can result in protein degradation and mute the performance of diagnostic protein tests on such samples. Thus, validation of the performance of the multiplexed detection of a bladder cancer biomarker panel in freshly voided urines will be required.

There is growing demand to integrate multiplex signatures to obtain favorable assay properties, such as reduced sample volume, decreased processing time, low cost analysis and low reagent consumption. Several multiplex protein services are available (*e.g.,* Sample Testing Services of Quansys Biosciences Inc., microplate-based; Aushon Biosystems, SearchLight Assays Services, microplate-based; Milliplex MAP, bead-based; and RayBiotech, Inc., slide-based). Several studies have reported that the multiplex ELISA procedures appear suitable and reliable for tissue lysate and serum [21,22]. While there are inherent, usually subtle differences between the various multiplex technologies, the overall technique is rapid, cost effective, and reliable. The reliability may be due in part to specific capture and detection antibody pairs that are optimized for the specific protein in question without cross-reactivity. The Q-Plex system combines powerful technology that allows the simultaneous determination of the expression levels of many proteins in biologic samples (*e.g.,* urine, blood, sputum, cerebrospinal fluid). The continued identification of biomarker proteins, which are associated with diagnosis or prognosis, will result in this technology being more widely adopted.

## Conclusion

Based on these encouraging preliminary data, we believe that the Q-Plex technology is a viable new platform that can be exploited to be a simple, yet accurate tool to quantitate a panel of diagnostic proteins in biologic specimens, in this case the detection of diagnostic bladder cancer biomarkers in voided urine samples. Importantly, this novel platform has acceptable inter assay variability, as well as very sensitive levels of detection. The platform can be readily implemented in a clinical laboratory and may be used as the basis for further work by incorporation of our entire bladder cancer-associated diagnostic signature.

## Methods

### Patients and specimen processing

Under MD Anderson Cancer Center Orlando Institutional Review Board approval and informed consent, prospectively collected voided urine samples (50 mL) were assigned a unique identifying number before immediate laboratory processing. Each urine sample was centrifuged at 600 × *g* 4°C for 5 min. The supernatant was decanted and aliquoted, and stored at -80°C in our genitourinary tissue bank prior to analysis. The genitourinary tissue bank was queried for suitable samples to analyze in the current study. Patients with known renal disease or documented renal insufficiency were not selected for inclusion in the current study. The study cohort consisted of 42 subjects with no previous history of urothelia carcinoma, gross hematuria, active urinary tract infection or urolithiasis (controls), and 31 subjects with newly diagnosed urothelial carcinoma. All 73 subjects a) had their urines analyzed by individual commercial ELISA kits for IL8, MMP9 and VEGFA [8] and b) had at least 5 mL of urine in the tissue bank for the current analysis. Frozen supernatant aliquots from the 73 subjects were shipped on dry ice to Quansys Bioscience, biotechnology company geared towards standardize or customize singleplex and multiplex ELISA assays, for analysis. Clinical information associated with these urine samples was collected. Data is reported using the STARD criteria [23]. Aliquots of urine supernatants were thawed and analyzed. The total protein concentration for each sample was determined via absorbance at 280 nm using a Nanodrop ND-1000 spectrophotometer (Thermo Scientific, Wilmington DE), and creatinine assays were performed on each sample for normalization with respect to urine volume [17].

### Q-plex multiplex assay

The Q-Plex technology is based on placement of immobilized capture antibody in 350–500 μm spots at the bottom of polypropylene 96-well plates to capture target proteins (for further details see http://www.quansysbio.com/assay-development/). Each spot is printed with a different analyte capture antibody, in this case against IL8, MMP9 and VEGFA. The sandwich immunoassay complex that forms is labeled via biotin-streptavidin with Horse Radish Peroxidase that generates light from a chemiluminescent substrate. Two internal control assays are designed within the assay to ensure integrity of results from each well through monitoring for the presence of interfering factors.

Thawed urine samples were handled on ice and diluted with Human Sample Dilution Buffer (Quansys), designed to reduce effects from heterophilic antibodies and other interferents [24]. Samples and standards (50 μl) were loaded onto the Q-Plex™ plate with a multichannel pipettor in order to reduce pipetting error and allowed to incubate for two hours. Laboratory personnel were blinded to final diagnosis. Subsequently, the sandwich immunoassay complex that forms was incubated with biotin-streptavidin/Horse Radish Peroxidase reagents for an additional two hours. Antigen standard curves were performed in duplicate diluting the antigen standard 1:3 for 11 points with a single negative point. Samples were diluted at ratios of 1 to 2 (sample to buffer) (50%), 1 to 20 (5%) and 1 to 200 (0.5%). Each dilution was loaded into three wells and measured in triplicate, a total of 9 wells per sample. Standard curves were constructed using Q-View Software™, which allows for the selection of multiple non-linear and linear equations to fit the standard curve. Optimal curve fits were determined by visual graph evaluation and comparison of Aikake’s information criteria (AIC) values [25]. A composite or stacked image composed of individual exposures of 30, 60, and 180 seconds with camera noise background subtraction was performed using Q-View Imager™. Levels of chemiluminescent units, or pixel intensity units were measured by Q-View Software™. The range of pixel intensity units is from 0 to 65536 (216 for a 16 bit image). Lower limits of quantification (LLOQ) were determined to be the lowest point of the 10-point positive standard curve where the back-fit regression values were within 20% of the known value.

### Commercial Enzyme-Linked Immunosorbent Assays (ELISA)

The levels of human Interleukin 8 (IL8, Cat # ab46032 Abcam, Cambridge, MA, USA), Matrix Metalloproteinase 9 (MMP9, Cat # DMP900 R&D Systems Inc., Minneapolis, MN, USA) and Vascular Endothelial Growth Factor A (VEGFA, Cat # 100663 Abcam, Cambridge, MA, USA) were monitored in voided urine samples, as previously reported [8].

### Data analysis

Nonparametric receiver operating characteristic (ROC) curves in which the value for sensitivity is plotted against false-positive rate (1-specificity) were generated for association of biomarkers with disease status and assay performance comparison. We defined a diagnostic test as positive or negative for bladder cancer detection by defining an optimal analyte concentration cutoff value (Youden index), selected to maximize the sum of the sensitivity and specificity [26,27]. Threshold cut-offs used were: IL8, 83 pg/ml; MMP9, 365,525 pg/ml; and VEGFA, 21,286 pg/ml. The relative ability of the combination of biomarkers to indicate bladder cancer was estimated by calculating the area under the ROC curves (AUC), and the sensitivity and specificity was defined by calculation of the Youden index. Statistical significance in this study was set at *p* < 0.05 and all reported *p* values were 2-sided. All analyses were performed using SAS software version 9.3 (SAS Institute Inc., Cary, NC).

## Abbreviations

IL8: Interleukin 8; MMP9: Matrix metallopeptidase 9; VEGFA: Vascular endothelial growth factor A; CV: Coefficient of variation; AUROC: Area under the receiver operating characteristic; PPV: Positive predictive value; NPV: Negative predictive value; ELISA: Enzyme-linked immunosorbent assay; STARD: STAndards for the Reporting of Diagnostic; AIC: Aikake’s information criteria; ROC: Receiver operating characteristic; AUC: Area under the concentration curve; LLD: Lower limits of detection; LLOQ: Lower limits of quantification; CI: Confidence interval; SD: Standard deviation; CLIA: Clinical laboratory improvement amendments.

## Competing interests

CJR and SG are officers at Nonagen BioScience Corp. YD, MM and GZ have no interests to disclose.

## Authors’ contributions

CJR – Study concept and design, drafting of manuscript, administrative support and funding. YD - Statistical analysis and drafting of manuscript. MM - Study design, statistical analysis and drafting of manuscript. GZ Sample collection, sample preparation and creation of database. SG Study concept and design, drafting of manuscript. All authors read and approved the final manuscript.
